# Ultrasound for diagnosing paediatric intestinal malrotation and midgut volvulus: a retrospective study in a single-centre high-suspicion cohort

**DOI:** 10.3389/fped.2026.1962837

**Published:** 2026-10-27

**Authors:** Fang Lin, Weicheng Wu, Hao Zhang, Yanyan Gao, Guangchao Zhang, Tianjiao Chang, Shiyu Gao, Song Sun, Yinghua Sun

**Affiliations:** 1 Department of Ultrasound, National Children’s Medical Center, Children’s Hospital of Fudan University, Shanghai, China; 2 Department of Pediatric Surgery, National Children’s Medical Center, Children’s Hospital of Fudan University, Shanghai, China

**Keywords:** children, intestinal malrotation, midgut volvulus, ultrasonography, whirlpool sign

## Abstract

**Background:**

Intestinal malrotation (IM) is a critical, surgery-requiring condition in children, potentially leading to life-threatening midgut volvulus. Prompt and accurate diagnosis is essential to prevent catastrophic outcomes. While the upper gastrointestinal series (UGI) has been a traditional diagnostic tool, ultrasonography (US) offers a nonradiation-based alternative.

**Objective:**

To evaluate the diagnostic value of ultrasound (US) in detecting IM and midgut volvulus in a high-clinical-suspicion paediatric cohort.

**Methods:**

A retrospective analysis was conducted on 143 children with high clinical suspicion of intestinal malrotation at the Children's Hospital between January 2017 and June 2024. All patients received a standardized US assessment, including evaluation of the superior mesenteric artery–superior mesenteric vein (SMA-SMV) relationship, the retroperitoneal position of the third portion of the duodenum (D3), and the presence of the “whirlpool sign.” Using surgical findings or clinical follow-up as the gold standard, the sensitivity, specificity, and accuracy of US for diagnosing intestinal malrotation and of the “whirlpool sign” for diagnosing midgut volvulus were calculated.

**Results:**

Among the 143 suspected cases, 110 (76.9%) were confirmed to have intestinal malrotation. The sensitivity and specificity of US for diagnosing IM were both 90.9%. Subgroup analysis indicated numerically higher diagnostic sensitivity and accuracy in neonates. Among the 110 patients with surgically confirmed IM, 78 (70.9%) were complicated with midgut volvulus. In this IM-confirmed subgroup, the whirlpool sign demonstrated a sensitivity of 93.6% (73/78) and a specificity of 93.8% (30/32) for identifying midgut volvulus (*P* < 0.001).

Analysis of the 13 discordant cases (3 false positives, 10 false negatives) revealed that false positives occurred in non-IM conditions such as mesenteric lymphadenitis, while all false negatives were surgically confirmed due to persistent high clinical suspicion.

**Conclusions:**

In a paediatric cohort with high clinical suspicion, US exhibits high diagnostic efficacy for both IM and midgut volvulus. The whirlpool sign serves as a critical indicator for emergency surgical decision-making. However, a negative US does not completely rule out IM and should be interpreted in conjunction with clinical findings.

## 1 Introduction

Intestinal malrotation (IM) is a common paediatric surgical emergency resulting from failure of the embryonic midgut to complete its normal 270° counterclockwise rotation. The neonatal period represents the peak incidence, with the classic clinical presentation being bilious vomiting. If not promptly managed, IM may be complicated by midgut volvulus, leading to intestinal ischemia, necrosis, and potentially life-threatening consequences (1, 2).

The incidence of IM is approximately 1 in 500 live births, while symptomatic cases occur in 1 in 6,000 neonates. Most symptomatic cases are diagnosed within the first month of life, with clinical manifestations typically including abdominal pain, vomiting, and failure to thrive (1, 3).

The traditional diagnostic modality for IM has been the upper gastrointestinal series (UGI); however, its diagnostic accuracy is suboptimal, with approximately 15% of examinations yielding inconclusive results (4). Because ultrasound (US) is radiation-free, portable, and highly repeatable, it has been increasingly utilized for the diagnosis of IM. Multiple studies and systematic reviews have demonstrated that US exhibits high diagnostic performance in identifying abnormalities in the superior mesenteric vessel relationship and the “whirlpool sign” (2, 5). Furthermore, the portability of US enables rapid performance across different hospital departments, thereby expediting the diagnostic process, particularly in emergency settings (6). An expert panel narrative review by Nguyen et al. (7) reported a pooled sensitivity of 94% and specificity of 100% for US in diagnosing IM, and a sensitivity of 92% and specificity of 99% for diagnosing midgut volvulus.

However, the existing evidence is largely derived from mixed-risk populations. In clinical practice, for paediatric cohorts with high pre-test probability—such as those referred to tertiary children's hospitals with classic symptoms like bilious vomiting—the precise diagnostic performance of US, particularly the characteristics of false-positive and false-negative cases, requires further clarification. Such analysis is crucial for optimizing clinical decision-making pathways and avoiding unnecessary surgery or missed diagnoses.

This study retrospectively analysed data from 143 paediatric patients who underwent standardized US examination for suspected IM at our institution between 2017 and 2024. Using surgical findings or clinical follow-up as the reference standard, we evaluated the diagnostic performance of US (including assessment of mesenteric vessel relationship, third portion of the duodenum position, and the “whirlpool sign”) for IM and midgut volvulus and conducted an in-depth analysis of discordant cases to provide more refined reference evidence for clinical practice.

## 2 Methods

### 2.1 Cohort

A total of 143 children who underwent US examination were admitted to our hospital between January 1, 2017, and June 30, 2024, with a high clinical suspicion of “intestinal malrotation”.

Two senior physicians independently evaluated the records based on predefined criteria for “high clinical suspicion” (described in detail below). A total of 180 patients were initially identified. Twenty-one patients were excluded due to incomplete clinical or imaging data, or loss to follow-up. Sixteen patients were excluded because they did not undergo an abdominal ultrasound examination. The patient selection process is illustrated in Figure 1 and was conducted as follows. A total of 143 paediatric patients with high clinical suspicion of intestinal malrotation were ultimately enrolled. All subjects underwent standardized ultrasonographic examinations prior to surgery. The final diagnosis was established based on the gold standard of operative exploration findings (118 cases) or clinical follow-up of at least 1 month (25 cases).

**Figure 1:**
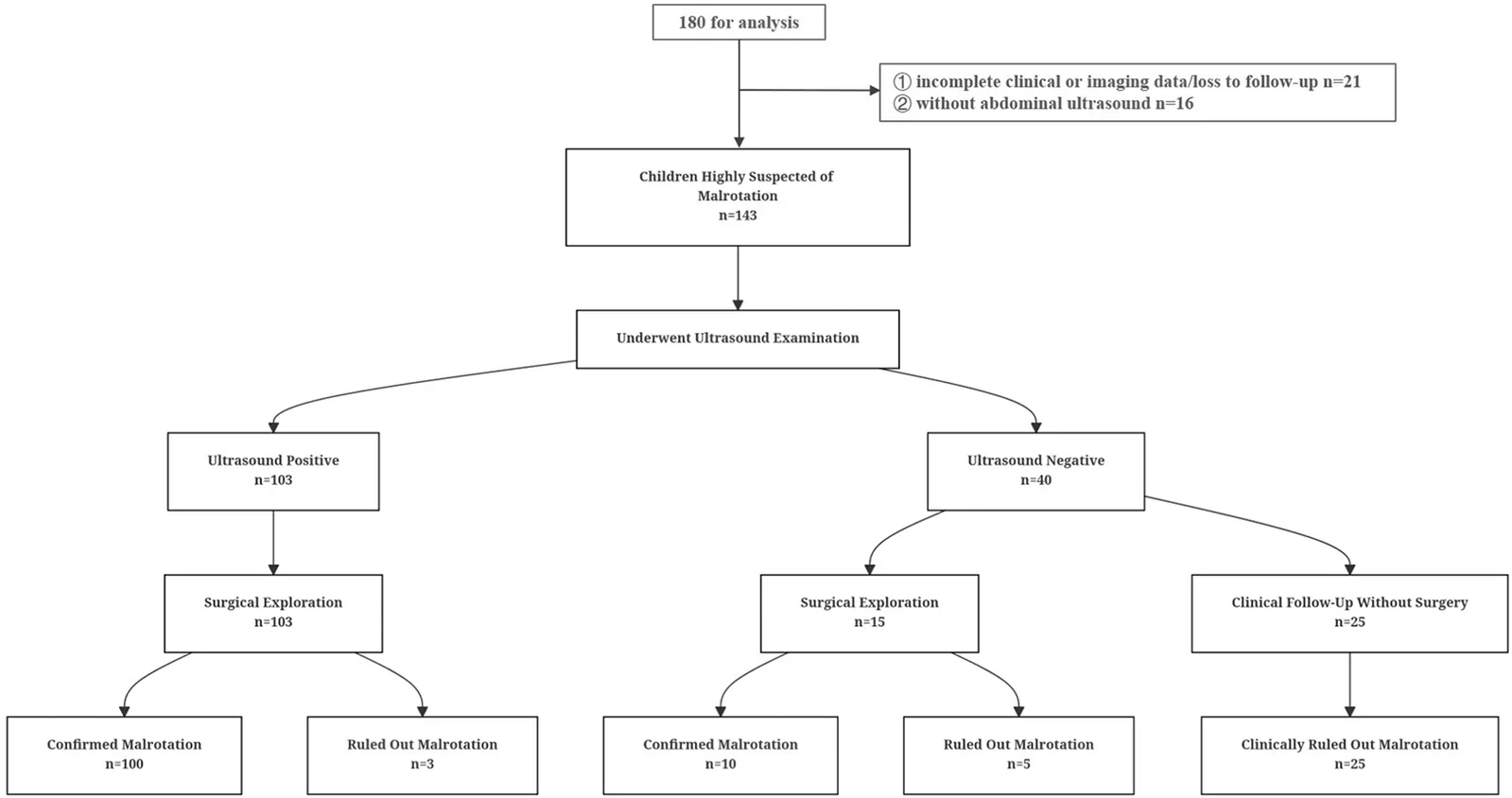
Diagnostic pathway for children with suspected intestinal malrotation. This flowchart illustrates the patient selection process, diagnostic workup, and final outcomes of children with suspected intestinal malrotation. A total of 180 patients were initially identified; 37 were excluded (21 with incomplete data or loss to follow-up, 16 without abdominal ultrasound). The remaining 143 patients underwent standardized US assessment. Patients with positive US results (*n* = 103) underwent surgical exploration directly. Among patients with negative US results (*n* = 40), 15 underwent surgical exploration because of strong clinical suspicion, while 25 were managed nonoperatively on the basis of clinical and other imaging findings. The final diagnosis was confirmed by surgical findings in the explored patients or clinical follow-up in the nonoperated patients. Ultimately, 110 patients were confirmed to have intestinal malrotation, and 33 patients were ruled out.

High clinical suspicion was based on one or more of the following findings (8, 9):

1. Bilious vomiting: the most important and characteristic warning sign, particularly in neonates and infants.
2. Acute onset of severe vomiting with abdominal pain: In older children, it may manifest as acute, unremitting abdominal pain and vomiting.
3. Abdominal signs: Including but not limited to abdominal distension, tenderness, guarding, or bloody stools, suggesting possible intestinal ischemia.
4. Imaging suggestions: The findings in some children were suggestive of duodenal obstruction (e.g., “double-bubble” sign) or other signs suspicious for intestinal obstruction on x-ray films performed at outside institutions.
5. Age at onset: This study focused on children, especially those who presented with the above symptoms during the neonatal period and infancy.

All enrolled children were selected according to the above clinical judgment. Among them, those with positive ultrasound findings (*n* = 103) or negative ultrasound findings but persistently high clinical suspicion (*n* = 15) underwent surgical exploration. The remaining patients with negative ultrasound findings who had clinical resolution of symptoms or did not meet surgical indications (*n* = 25) were followed up clinically for at least 1 month. The final diagnosis was established based on surgical findings (118 cases) or clinical follow-up (25 cases) as the reference standard.

### 2.2 Instruments and methods

All US examinations were performed by experienced US physicians or US technicians under the supervision of attending physicians, and the examinations followed a structured protocol based on expert consensus (7). Examinations were conducted using Philips EPIQ 7 (Philips Healthcare), GE LOGIQ E11, or SAMSUNG RS80A ultrasound systems equipped with high-frequency linear array probes (5–12 MHz). The use of high-frequency probes is standard in pediatric imaging for optimal spatial resolution.

1. Standard abdominal survey: A comprehensive abdominal examination was performed first.
2. Evaluation of Superior Mesenteric Vessels (SMV-SMA): The probe, placed in a transverse or slightly oblique section at the upper abdomen, was used to display the SMA, SMV, and abdominal aorta (Ao) at the level of the pancreas. The normal relationship is that the SMV is located to the right of the SMA (Figure 2a) (16). An abnormal relationship, where the SMV root is located to the left of or directly anterior to the SMA root (Figure 3-a), was considered a positive sign of malrotation (8).
3. Duodenal Assessment: The duodenum was traced distally from the pylorus, with emphasis on confirming whether the third portion (D3) traversed the retroperitoneal space between the aorta and the SMA (Figure 2b). Anterior positioning of D3 relative to the SMA was considered anomalous (Figure 3b) (16).
4. Assessment of the “whirlpool sign”: Using a graded compression technique, transverse sections along the course of the SMA were observed. A mass with alternating hypoechoic stripes was observed, with the SMA cross-section centered and the bowel loops, mesentery, and SMV located peripherally. Upon continuous transverse scanning from the SMA root toward its distal end, the peripheral bowel loops, mesentery, and SMV were observed to rotate clockwise around the long axis of the SMA, forming the “whirlpool sign” (Figure 3-c). On color Doppler, this appeared as a central pulsatile flow dot from the SMA, surrounded by a ring of red and blue flow from the SMV rotating clockwise (Figure 3-d). A clockwise whirlpool sign was considered a positive US finding for midgut volvulus (10, 11).

**Figure 2:**
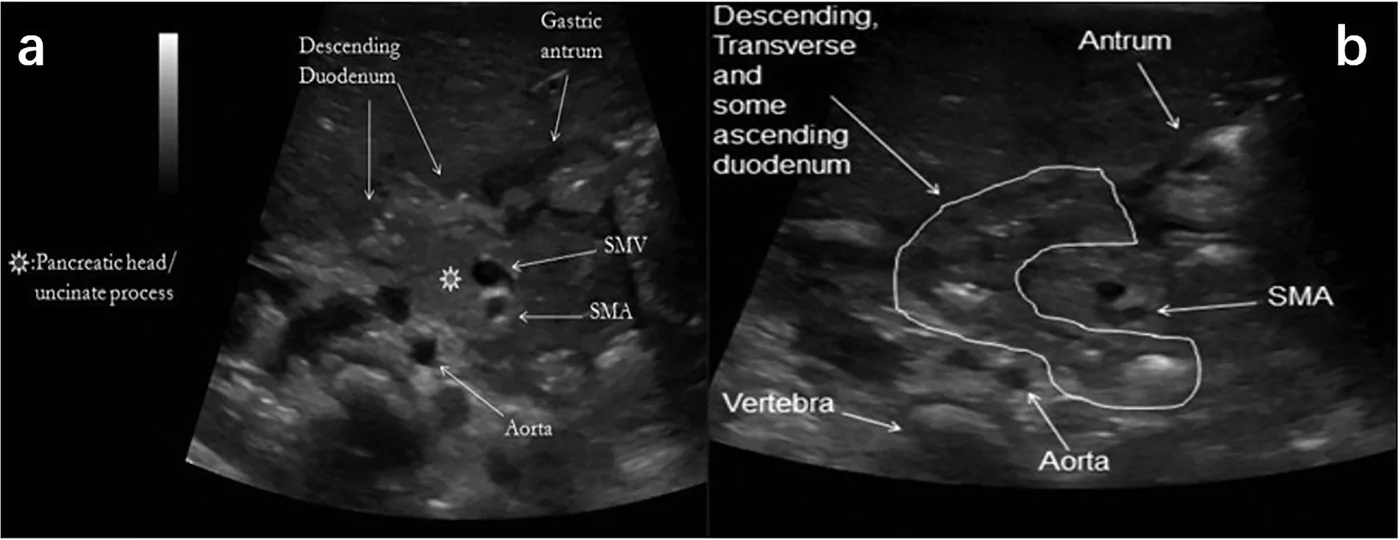
Normal sonographic anatomy of the mesenteric vessels and duodenum. **(a)** Transverse ultrasound image showing the superior mesenteric vein (SMV), superior mesenteric artery (SMA), aorta, and surrounding anatomical structures. **(b)** Ultrasound image demonstrating the normal course of the transverse duodenum between the SMA and aorta; the duodenal course is outlined in white. Images reproduced from Figures 3 and 4, respectively, of Herliczek et al. (2011), under the Creative Commons Attribution License stated on the publisher's article page. © 2011 Herliczek TW. The images have been assembled and labelled as panels **(a)** and **(b)** (16).

**Figure 3:**
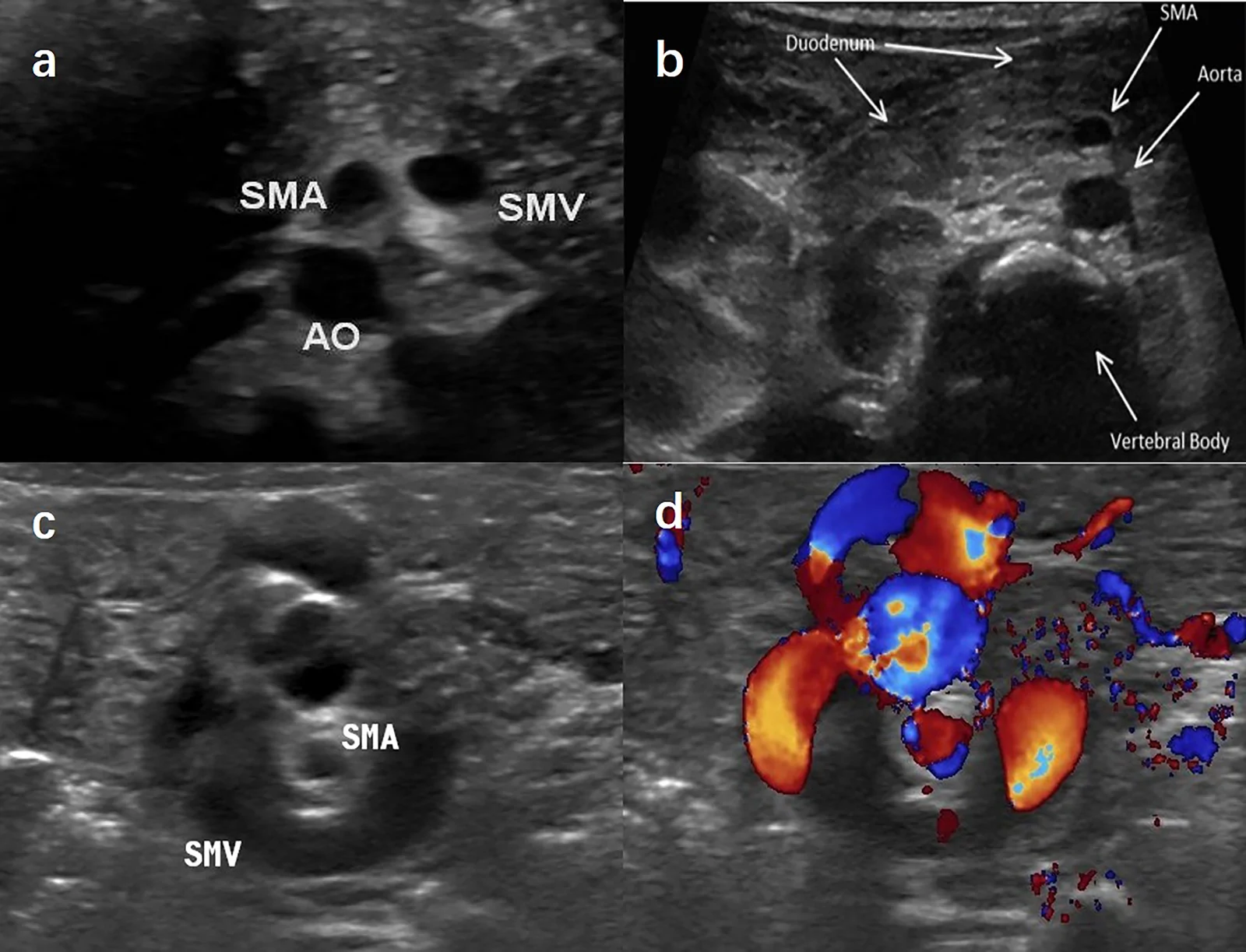
**(a)** abnormal relationship of the superior mesenteric vessels in a child with intestinal malrotation. Transverse grayscale US image of the upper abdomen showing the superior mesenteric vein (SMV) abnormally located to the left of the superior mesenteric artery (SMA); **(b)** Transverse ultrasound image showing an abnormal course of the transverse duodenum anterior to the superior mesenteric artery (SMA), rather than between the SMA and aorta (16); **(c,d)** Ultrasonographic “whirlpool sign” in a child with midgut volvulus. **c:** Transverse grayscale US image of the upper abdomen, using graded compression, showing the mesentery and vessels whirling around the root of the SMA. **d:** Colour Doppler image at the same location clearly demonstrating the vortex formed by the SMV and its branches rotating around the SMA. This finding provides direct evidence for the diagnosis of midgut volvulus. Panel **(b)** is reproduced from Figure 11 of Herliczek et al. (2011), under the Creative Commons Attribution License stated on the publisher's article page. © 2011 Herliczek TW.

### 2.3 Image analysis and diagnostic criteria

Data analysis utilized the original US reports generated during the patient’s initial visit, reflecting real clinical practice.

A positive US diagnosis for intestinal malrotation required meeting either of the following criteria:

Criterion 1: Demonstration of abnormal relative positions of the SMV and SMA (SMV located to the left of or directly anterior to the SMA).
Criterion 2: An intraperitoneal course of D3, positioned anterior to the SMA, rather than its normal retroperitoneal location between the aorta and the SMA.
Criterion 3: Presence of the “whirlpool sign”.

The diagnosis of intestinal torsion in ultrasonography relies exclusively on the presence of the “whirlpool sign.”

### 2.4 Assessment of interobserver agreement

To evaluate the consistency of ultrasonographic diagnoses in a real-world clinical setting, we assessed the interobserver agreement among the sonographers. The four attending sonographers who performed the majority (>90%) of the examinations in this study were included in this analysis. For patients who had independent assessments documented by more than one of these primary sonographers (e.g., through a formal second-opinion process or concurrent evaluation during teaching sessions), the final diagnostic impressions (positive or negative for intestinal malrotation) as recorded in the original clinical reports were compared. To calculate the overall agreement, the four attending sonographers were grouped into all possible evaluator pairs (6 pairs). Cohen's kappa (*κ*) was calculated for each pair based on their independently documented assessments, and the overall *κ* value was derived as the mean of these pairwise *κ* estimates. The resulting overall *κ* was 0.78 (95% CI: 0.68–0.88), indicating substantial agreement. This approach ensured that the agreement estimate reflected the consistency of interpretations across the attending sonographers in routine practice, rather than being dependent on any single pair of evaluators.

### 2.5 Statistical analysis

Statistical analysis was performed using SPSS software (version 26.0; IBM Corp.). Continuous data with a normal distribution are presented as the mean ± standard deviation, while nonnormally distributed data are expressed as the median and interquartile range. Categorical variables are described as frequencies and percentages. Using surgical findings as the reference standard, the diagnostic performance of ultrasonography was evaluated by calculating the sensitivity, specificity, accuracy, and corresponding 95% confidence intervals (CIs). A value of *P* < 0.05 was considered to indicate statistical significance.

## 3 Results

### 3.1 Study population and clinical characteristics

This study included a total of 143 children with a high clinical suspicion of intestinal malrotation, 118 of whom underwent surgical exploration. Among those who underwent surgery, 110 (93.2%) were confirmed to have intestinal malrotation intraoperatively and were included in the final analysis. As shown in the Table 1, males predominated (63.6%), and the vast majority (71.3%) were neonates (≤28 days). With respect to the mode of onset, the vast majority of the children (86.4%) presented acutely, with vomiting being the most common initial symptom (69.2%).

**Table 1:** Demographics, clinical presentations, and US diagnostic performance in the overall cohort and across different age subgroups.

	No. (%)		
Characteristic	Overall (*n* = 143)	Neonates (*n* = 102)	Non-neonates (*n* = 41)
Sex (Male: Female)	(91/52)	(69/33)	(22/19)
Mean age (days)	471.7	4.02	1635.2
Initial clinical manifestation			
Emesis	99 (69.2)	81 (79.4)	18 (43.9)
Jaundice	30 (21.0)	10 (9.8)	20 (48.8)
Others	14 (9.8)	11 (10.8)	3 (7.3)
US results			
Positive	103	77	26
Negative	40	25	15
Surgical result or clinical follow-up			
Intestinal malrotation	110	81	29
No malrotation	33	21	12
Sensitivity (95% CI)	90.9 (84.1−95.0)	92.6 (85.0–96.5)	86.2 (69.4–94.5)
Specificity (95% CI)	90.9 (76.4–96.9)	90.5 (71.1–97.3)	91.7 (64.6–98.5)

Using surgical findings or clinical follow-up data as the gold standard, the overall diagnostic performance of US for intestinal malrotation is shown in Table 1. Among the 143 suspected cases, US demonstrated a sensitivity of 90.9% (100/110), a specificity of 90.9% (30/33), and an overall accuracy of 90.9% (Table 2).

**Table 2:** Overall efficacy of ultrasonography in diagnosing intestinal malrotation.

US Result	Surgical Result or Clinical Follow-up (No Surgery)	
	Intestinal Malrotation (*n* = 110)	No Malrotation (*n* = 33)
Positive (*n* = 103)	100	3
Negative (*n* = 40)	10	30

Among the 143 enrolled patients, 25 did not undergo surgical exploration and were managed conservatively. The clinical characteristics and follow-up outcomes of these patients are summarized in Supplementary Table 1. The median follow-up duration was 6 months (range: 1-18 months). Additional imaging, including upper gastrointestinal series (*n* = 10) or abdominal radiography (*n* = 12), was performed in 22 patients (88.0%), all of which showed no evidence of intestinal malrotation or obstruction. The remaining 3 patients (12.0%) had spontaneous resolution of symptoms shortly after admission and were monitored clinically without further imaging. At the end of follow-up, 23 patients (92.0%) had complete symptom resolution, and the remaining 2 patients (8.0%) experienced only mild intermittent symptoms that did not require surgical intervention. On the basis of these comprehensive clinical and imaging assessments, intestinal malrotation was considered adequately excluded in all 25 patients (Supplementary Table 1).

### 3.2 Age distribution in neonates and age subgroup analysis of diagnostic efficacy

The age distribution at presentation for the neonates with confirmed IM (*n* = 81) is shown in Figure 4. The mean age of the neonates was 4.02 ± 5.80 days. The distribution was markedly right skewed, indicating that most infants became symptomatic and presented for medical attention very early in life, with the highest concentration occurring within the first day after birth. The average time from symptom onset to presentation was 3.2 days. To further evaluate the diagnostic performance of US across different age groups, an age subgroup analysis was performed (Table 3). In the neonatal group (*n* = 102), US achieved a sensitivity of 92.6%, a specificity of 90.5%, and an accuracy of 92.2%. In the nonneonatal group (*n* = 41), the sensitivity was 86.2%, the specificity was 91.7%, and the accuracy was 87.8%.

**Figure 4:**
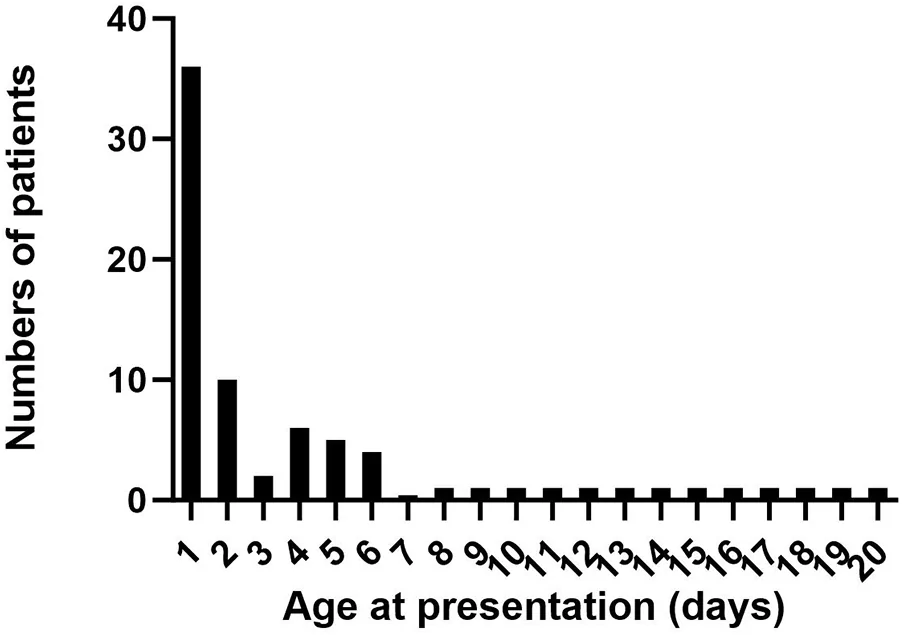
Histogram showing the age at presentation for the neonatal group with confirmed intestinal malrotation (*n* = 81). The distribution is markedly right skewed, indicating that most infants became symptomatic and presented for medical attention very early in life.

**Table 3:** Age subgroup analysis of ultrasonography for diagnosing intestinal malrotation (based on all suspected children, *n* = 143).

Study Population	US Result	Surgical Result or Clinical Follow-up (No Surgery)	
		Intestinal Malrotation (*n* = 110)	No Malrotation (*n* = 33)
Neonates (*n* = 102)	Positive (*n* = 77)	75	2
	Negative (*n* = 25)	6	19
Nonneonates (*n* = 41)	Positive (*n* = 26)	25	1
	Negative (*n* = 15)	4	11

### 3.3 Diagnostic value of the “whirlpool sign” for midgut volvulus

Among the 110 surgically confirmed IM cases, 78 (70.9%) were complicated by midgut volvulus. The diagnostic performance of the ‘whirlpool sign’ for identifying volvulus within this IM-confirmed subgroup is shown in Table 4. It demonstrated exceptionally high diagnostic value, with a sensitivity of 93.6% (73/78), a specificity of 93.8% (30/32), and an accuracy of 93.6% (103/110) (Table 4). The association between the presence of the “whirlpool sign” and surgically confirmed volvulus was statistically significant (*P* < 0.001).

**Table 4:** Diagnostic value of the “whirlpool sign” for midgut volvulus in 110 children with confirmed intestinal malrotation.

US Result	Surgical Result	
	IM with Volvulus (*n* = 78)	IM without Volvulus (*n* = 32)
Whirlpool sign present (*n* = 75)	73	2
Whirlpool sign absent (*n* = 35)	5	30

US, ultrasonography; IM, intestinal malrotation.

The interobserver agreement for the final US diagnosis of intestinal malrotation among the primary attending sonographers was substantial, with a Cohen’s kappa value of 0.78 (95% CI: 0.68–0.88).

### 3.4 Analysis of cases with diagnostic discrepancies

In this study, US findings were inconsistent with surgical results in 13 paediatric patients, including 3 false-positives and 10 false-negatives (Table 5). Among the 3 false-positive cases, 2 were diagnosed as malrotation because SMV was located directly anterior to the SMA; intraoperative findings confirmed mesenteric lymphadenitis in one case and normal anatomical variation in the other. The remaining false-positive case showed a suspected whirlpool sign on ultrasound, which was intraoperatively confirmed to be caused by compression from a duplication cyst.

**Table 5:** Detailed analysis of cases with discordance between ultrasound diagnosis and surgical findings.

Case type	Number of cases	Sonographic findings	Final diagnosis
False positive	3	Abnormal SMA/SMV relationship; suspected whirlpool sign	Mesenteric lymphadenitis; normal variant; enteric duplication cyst
False negative	10	Normal SMA/SMV relationship, no whirlpool sign	All confirmed as intestinal malrotation
Other non-malrotation diseases surgically confirmed despite negative US findings	5	US negative	Duodenal atresia; duodenal diaphragm; adhesive intestinal obstruction; Ladd bands

In the 10 false-negative cases, US demonstrated a normal SMV/SMA relationship and no whirlpool sign in all patients. All of these patients underwent surgical exploration due to strong clinical indications, including persistent bilious vomiting (10/10), and malrotation was confirmed intraoperatively in each case.

Five patients with negative US findings underwent surgical exploration because of persistent high clinical suspicion (bilious vomiting, obstructive findings on abdominal x-ray, etc.). Final diagnoses included duodenal atresia (2 cases), duodenal diaphragm (1 case), adhesive intestinal obstruction (1 case), and Ladd bands without malrotation (1 case) (Table 5).

## 4 Discussion

This study, based on clinical and ultrasonographic data from 143 suspected cases, confirms the significant value of ultrasonography in the diagnosis of intestinal malrotation and its complication—midgut volvulus—in children. Our results show that, on the basis of the entire cohort of suspected patients, US achieved an overall sensitivity and specificity of 90.9% each, with an overall accuracy of 90.9%, aligning with the findings from the expert panel narrative review by Nguyen et al. and further validating the utility of ultrasonography as a first-line imaging modality (7, 12–14). However, the high diagnostic efficacy observed in this study is partially attributable to the relatively high pre-test probability. In a general paediatric population with vomiting, where disease prevalence is lower, the positive predictive value (PPV) would decrease accordingly, whereas the negative predictive value (NPV) would increase. Nevertheless, this does not imply that a negative ultrasound result can completely rule out IM. For an individual child with persistently high clinical suspicion, a negative ultrasound finding should still be interpreted with caution and should not serve as the sole basis for deferring surgical intervention.

It should be noted that the diagnostic performance analysis of the whirlpool sign was conducted within the subgroup of patients with surgically confirmed IM. Therefore, the reported sensitivity and specificity reflect the ability of this sign to identify volvulus among patients already diagnosed with IM, rather than its screening performance for volvulus in the entire cohort of children presenting with suspected malrotation. A key finding of this study is the near-perfect discriminatory power of the “whirlpool sign” for diagnosing midgut volvulus, with a sensitivity and specificity both exceeding 93% and a statistically significant association with surgical findings (*P* < 0.001). This aligns closely with the established pathophysiology of IM: a narrow mesenteric root predisposes the midgut to volvulus around the SMA, producing the characteristic “whirlpool sign” on US as direct evidence of midgut volvulus. Our results highlight the critical role of this sign in rapidly identifying cases requiring urgent surgical intervention, which is paramount for preventing intestinal necrosis and improving patient outcomes (11, 15). The low number of false positives (*n* = 2) and false negatives (*n* = 5) for the “whirlpool sign” in the diagnosis of volvulus further underscores its practical utility in distinguishing complicated cases.

In contrast to the traditional upper gastrointestinal series (UGI), which requires ionizing radiation and is less portable, US provides a non-invasive, radiation-free alternative that can be performed at the bedside. These inherent advantages of US, as well documented in the literature, make it particularly suitable for neonatal imaging (11). Our study delineates the unique clinical profile of the cohort: Neonates (≤28 days) constituted a high proportion (71.3%), the vast majority (86.4%) presented acutely, and vomiting was the predominant symptom. This profile is consistent with the anatomical peculiarities of the infant digestive system, making the neonatal period a high-risk window for IM and related complications (11).

In the age subgroup analysis, we observed a notable trend: the sensitivity and accuracy of US for diagnosing IM were numerically greater in the neonatal group (92.6% and 92.2%, respectively) than in the nonneonatal group (86.2% and 87.8%, respectively). This trend might reflect inherent characteristics of the neonatal period, such as earlier presentation due to typical symptoms, less intestinal gas, thus facilitating imaging, and thinner abdominal walls, providing better acoustic windows. However, this trend must be interpreted cautiously. Statistical testing indicated that the differences in sensitivity and specificity between the neonatal and nonneonatal groups were not statistically significant (*p* > 0.05). Therefore, while a numerical advantage was observed, the current data cannot confirm that the diagnostic efficacy of US is significantly superior in neonates to that in older children. Future studies with larger sample sizes, particularly those that include more nonneonates, are warranted to validate and further explore these preliminary findings.

From a clinical application perspective, we recommend US as a valuable initial imaging modality for children with suspected IM. Particularly for neonates and infants presenting with vomiting, early implementation of US can facilitate the timely identification of malrotation and volvulus, preventing delays in surgical intervention. Furthermore, the US examination should be standardized, including systematic evaluation of the SMV–SMA relationship, active identification of the “whirlpool sign”, and identification of indirect signs such as duodenal dilation.

This study has several limitations. First, the reference standard was not identical for all patients: 25 cases (17.5%) were classified based on clinical follow-up rather than surgical pathology, which may have introduced partial verification bias. Nevertheless, all patients in this subgroup achieved complete symptom resolution during follow-up (median 6 months), and the clinical outcomes were unequivocal; thus, the impact on the overall diagnostic performance estimates is likely to be modest, although it should still be considered when interpreting the results. Second, its single-center, retrospective design and relatively small sample size, particularly in the nonmalrotation group, are limitations. Third, the most significant limitation pertains to the high disease prevalence cohort (77%). Under such conditions, the calculated positive predictive value (PPV) is relatively high, whereas the negative predictive value (NPV) is 75% (30/40). In a general paediatric population with a lower pre-test probability, while the NPV would be higher, the absolute number of false-negative cases could still be clinically meaningful. Therefore, relying solely on US to exclude IM carries a risk of missed diagnosis, particularly in children with persistent clinical suspicion. Fourth, from a technical perspective, although a standardized protocol including duodenal evaluation was implemented, some early cases might have been included in this retrospective study without a systematic assessment of the D3 segment, which could have influenced diagnostic performance to some extent.

In conclusion, this study confirms that ultrasonography is a highly effective and noninvasive imaging method for diagnosing intestinal malrotation and midgut volvulus in children. Its diagnostic value is strongly supported by the evaluation encompassing clinically followed cases within the entire cohort. It is particularly important to emphasize that the “whirlpool sign” is an extremely reliable indicator of volvulus and can serve as a key basis for decision-making regarding emergency surgery.

## Data Availability

The original contributions presented in the study are included in the article/Supplementary Material, further inquiries can be directed to the corresponding authors.
