# Supplementary material for: Ultrasound for diagnosing paediatric intestinal malrotation and midgut volvulus: a retrospective study in a single-centre high-suspicion cohort
Source: Front Pediatr. 2026 Oct 27;14:1962837. doi: 10.3389/fped.2026.1962837 (PMC13748241; doi:10.3389/fped.2026.1962837)
Supplement: Supplementary file 1 [file Table1.docx]

**Supplementary Table 1. Clinical characteristics and follow-up outcomes of the 25 non-operatively managed patients**

| **Characteristic** | **No. (%)** |
| --- | --- |
| **Sex (Male: Female)** | 16/9 |
| **Age group (days)** |  |
| Neonates (≤28 days) | 16 (64.0) |
| Non-neonates (>28 days) | 9 (36.0) |
| **Initial clinical manifestation** |  |
| Emesis | 20 (80.0) |
| Jaundice | 3 (12.0) |
| Others | 2 (8.0) |
| **Additional imaging performed** |  |
| Upper gastrointestinal series (UGI) | 10 (40.0) |
| Abdominal radiograph only | 12 (48.0) |
| No additional imaging (symptoms resolved spontaneously) | 3 (12.0) |
| **Follow-up modality** |  |
| Outpatient clinic visit | 15 (60.0) |
| Telephone interview | 10 (40.0) |
| **Clinical outcome** |  |
| Complete resolution of symptoms | 23 (92.0) |
| Mild intermittent symptoms without need for surgery | 2 (8.0) |
| **Basis for excluding intestinal malrotation** | Complete symptom resolution + normal UGI (n=10); normal radiograph findings with symptom resolution (n=12); spontaneous symptom resolution with stable follow-up (n=3) |
